# Gastrodoudenal Embolization: Indications, Technical Pearls, and Outcomes

**DOI:** 10.3390/jcm7050101

**Published:** 2018-05-02

**Authors:** Gokhan Kuyumcu, Igor Latich, Rulon L. Hardman, Gabriel C. Fine, Rahmi Oklu, Keith B. Quencer

**Affiliations:** 1 Department of Radiology and Biomedical Imaging, Section of Interventional Radiology, Yale University School of Medicine, New Haven, CT 06519, USA; Gokhan.Kuyumcu@ascension.org (G.K.); IGOR.LATICH@YALE.EDU (I.L.); 2 Division of Interventional Radiology, University of Utah Department of Radiology, Salt Lake City, UT 84108, USA; Rulon.Hardman@hsc.utah.edu (R.L.H.); Gabriel.Fine@hsc.utah.edu (G.C.F.); 3 Department of Vascular and Interventional Radiology, Minimally Invasive Therapeutics Laboratory, Mayo Clinic, Phoenix, AZ 85054, USA; Oklu.Rahmi@mayo.edu

**Keywords:** gastroduodenal artery, upper GI bleed embolization, pseudoaneurysm

## Abstract

The gastroduodenal artery (GDA) is frequently embolized in cases of upper GI bleed that has failed endoscopic therapy. Additionally, it may be done for GDA pseudoaneurysms or as an adjunctive procedure prior to Yttrim-90 (Y90) treatment of hepatic tumors. This clinical review will summarize anatomy and embryology of the GDA, indications, outcomes and complications of GDA embolization.

## 1. Introduction

Upper gastrointestinal (GI) bleeding is a common and potentially deadly medical condition with an estimated mortality of 10% [1]. The majority of upper GI bleeds are caused by peptic ulcer disease, which are most commonly located between the gastric antrum and third portion of the doudenum. Endoscopic and supportive medical therapies are the first line therapies, as they are highly effective. However, approximately 10% of all patients either continue to bleed or experience re-bleeding within 48 h of the endoscopic treatment. While surgical therapy has historically been considered the next line of treatment for upper GI refractory bleeding to medical and endoscopic managment, endovascular embolization has now become the next line therapy [2]. The gastrodoudenal artery (GDA) is the most common target for embolization as it supplies the territory that is most commonly affected by peptic ulcers. Additionally, GDA embolization may be performed prior to the Yttrium-90 (Y-90) administration or in cases of pseudoaneurysms [3]. In this paper, we review the indications, techniques, and outcomes of endovascular embolization of the GDA

## 2. Anatomy and Embryology of Gastroduodenal Artery

The GDA, like other mesenteric arteries, originate from the embryological descending aorta, which is in turn developed from the primitive ventral segmental arteries [4]. Most of the segmental arteries regress. The 10th segmental artery does not regress and gives rise to the celiac artery, which supplies the foregut [4]. Variability during regression and development leads to a variant anatomy of visceral arteries, including the GDA [4,5].

The GDA arises from the common hepatic artery. Lateral to the GDA origin, the common hepatic artery becomes the proper hepatic artery. The variable origins of the GDA include the superior mesenteric artery, the left hepatic artery, and the right hepatic artery [6]. It courses through upper margin of the pancreas for a variable distance. The GDA then descends vertically between the anterior surface of the pancreas and the posterior aspect of the duodenum. Posterior to the first part of the duodenum, the GDA gives rise to the supraduodenal artery. The GDA then bifurcates into the right gastroepiploic artery, which runs in the greater omentum along the greater curvature of the stomach and the superior pancreaticoduodenal artery. This latter branch divides into anterior and posterior branches, which encircle the pancreatic head. The posterior division anastomoses with the inferior pancreaticoduodenal artery (IPDA), which arises from the superior mesenteric artery [6,7]. The GDA supplies the gastric antrum, proximal duodenum, and the head of the pancreas.

## 3. GDA Embolization in Gastrointestinal Bleeding

Gastrointestinal bleeding is a significant public health issue, with an annual estimated direct cost to the health care system of five billion dollars [8]. Patients with upper GI bleed will typically manifest with hematemesis, melena, hypotension, tachycardia, and acute blood loss anemia. The severity of the symptoms will depend on the briskness of the bleeding and underlying patient conditions. The first step in treatment of upper GI bleeding is patient stabilization, including the placement of two large bore IVs, fluid resuscitation, and transfusion.

Upper GI bleed can be variceal or non-variceal. The former is a sequela of portal hypertension with treatment options including endoscopic banding, and/or sclerotheraphy, transjugular intrahepatic portalsystemic shunt (TIPS) placement +/− embolization, and/or balloon occluded retrograde transvenous obliteration/variants (BRTO) [9,10]. The causes of nonvariceal UGIB include peptic ulcer disease, Mallory–Weiss tears, erosive gastritis, duodenitis, esophagitis, malignancy, and angiodysplasias [1,11]. Of these, peptic ulcer disease is the most common cause.

Historically, the primary etiology of peptic ulcers was the *Helicobacter pylori* (*H. pylori*) infection. With the advent of the improved detection and treatment of *H. pylori*, non-steroidal anti-inflammatory drugs (NSAIDs) have become the most common cause [12]. These drugs cause ulcers by both local and systemic prostaglandin inhibition [1,12].

Acid suppression with intravenous proton pump inhibitors (IV PPI) should be given to all patients with upper GI bleeding. A typical dose and drug is esomeprazole 40 mg twice daily. When an endoscopy shows a high risk ulcer, therapy should be continued for at least 72 h [13]. While the H2 receptor antagonist (e.g., cimetidine) can be useful in the setting of gastric ulcers, they have shown no benefit in patients with duodenal ulcers [14].

**Figure 1:**
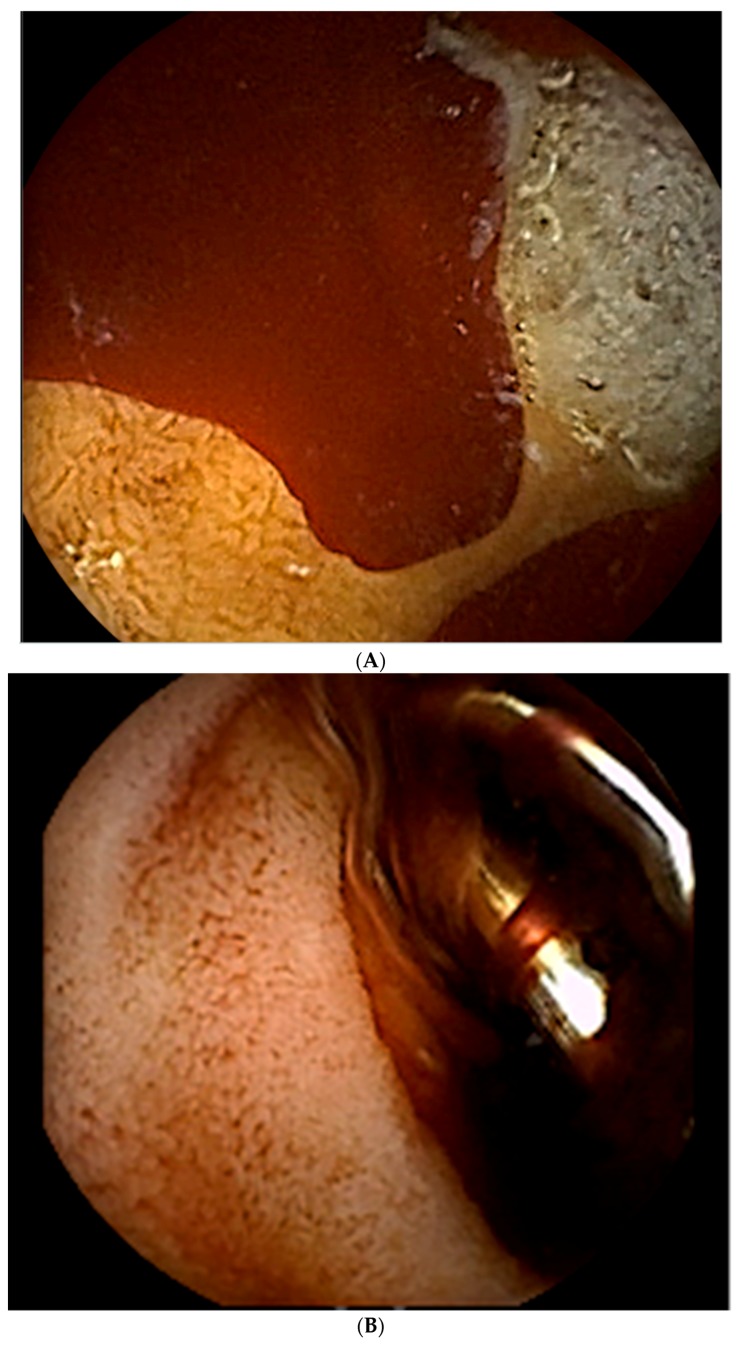

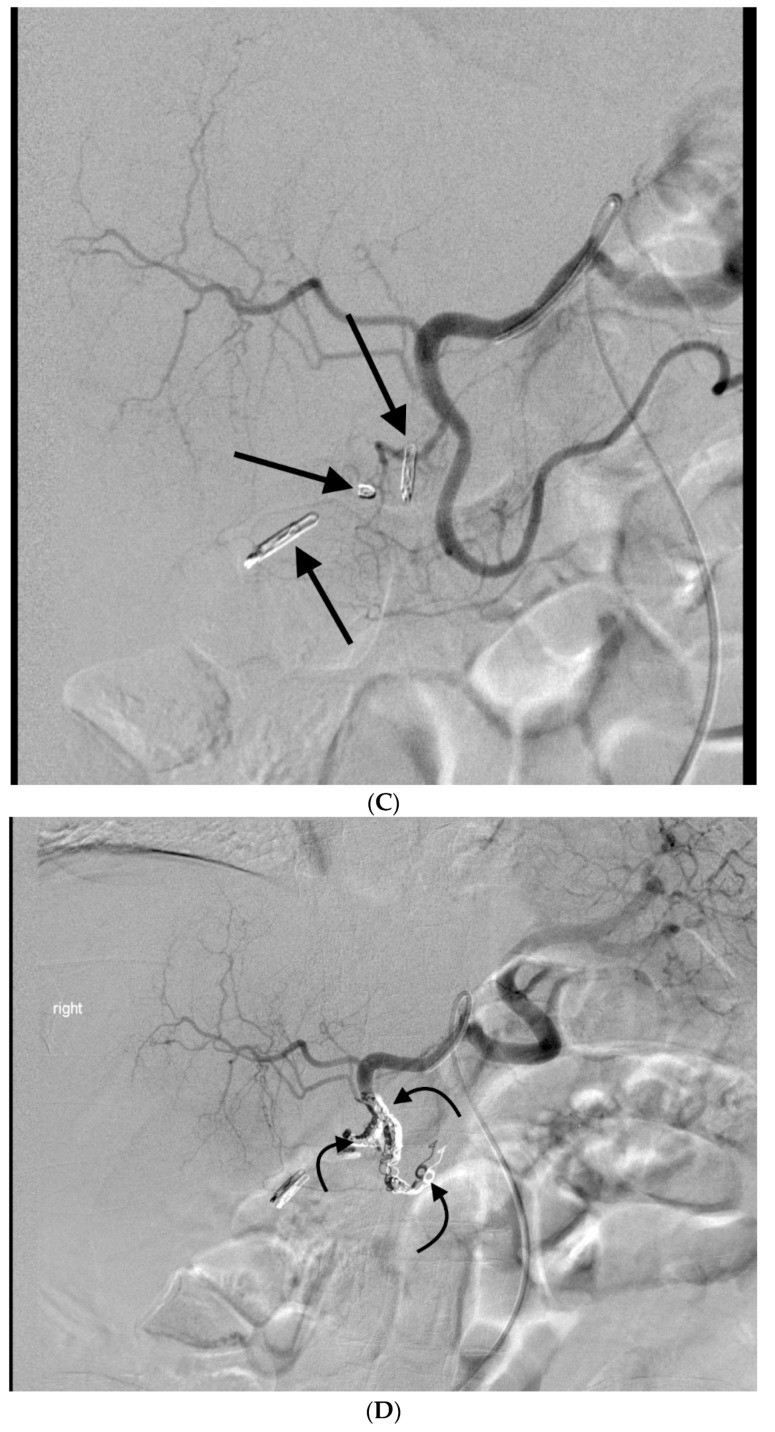
This 72 year old man presented with hematemesis and melena. (**A**) He underwent an upper endoscopy, at which time blood was seen in the duodenal bulb with two cratered oozing ulcers present. The largest ulcer was 10 mm in diameter. (**B**) The ulcers were treated with an injection of 4cc of 1:10,000 epinephrine, followed by the placement of hemostatic clips. (**C**) While hemostasis was achieved at the time of endoscopy, the patient rebled 3 h later. An angiogram shows multiple endoscopic clips (black arrows) in the duodenum, a region supplied by the gastrodoudenal artery (GDA). These clips helped to guide the angiographer to the location of the bleed. The (**D**) coil (curved black arrows) and gelfoam embolization was performed on the GDA.

Endoscopy is the mainstay in treatment of duodenal bleeding. Prior to endoscopy, a prokinetic agent (e.g., erythromycin or metoclopramide) is recommended in order to enhance the visualization of the underlying abnormality [15]. Various potential endoscopic treatments exist, including injection (epinephrine, sclerosants, fibrin, and cyanoacrylate glue), hemostatic clipping, thermal coagulation, and argon plasma coagulation. The results on the comparative efficacy of the different modalities are varied [16,17]. A combination of modalities is most often used (Figure 1). Hemostatic clips work by compression of the bleeding vessel. They typically slough off with the mucosa within a few weeks of placement. As far as thermal therapy goes, argon plasma coagulation is preferred, as the depth of heating is only 2–3 mm compared with the possible deeper burn of thermal coagulation. Overall, the combination of PPI and endoscopic therapy leads to the durable cessation of bleeding in over 90% of cases [13]. Even when endoscopic therapy fails, it maintains its utility by helping to discern the vascular territory from which the bleeding arises. The hemorrhage in the body and fundus of the stomach arises from the left gastric artery and bleeding from the pylorus, and the 1st through 3rd portions of the duodenum arise from the GDA [2].

Traditionally, the total and subtotal gastrectomies, truncal vagotomy and pyloroplasty, Billroth II gastrectomy, excision and under-running of ulcer, and even GDA ligation, has been considered as the next treatment modality. With the recent advances of transarterial embolization, however, endovascular therapy has supplanted surgery as the next step in treatment [18,19,20].

**Figure 2:**
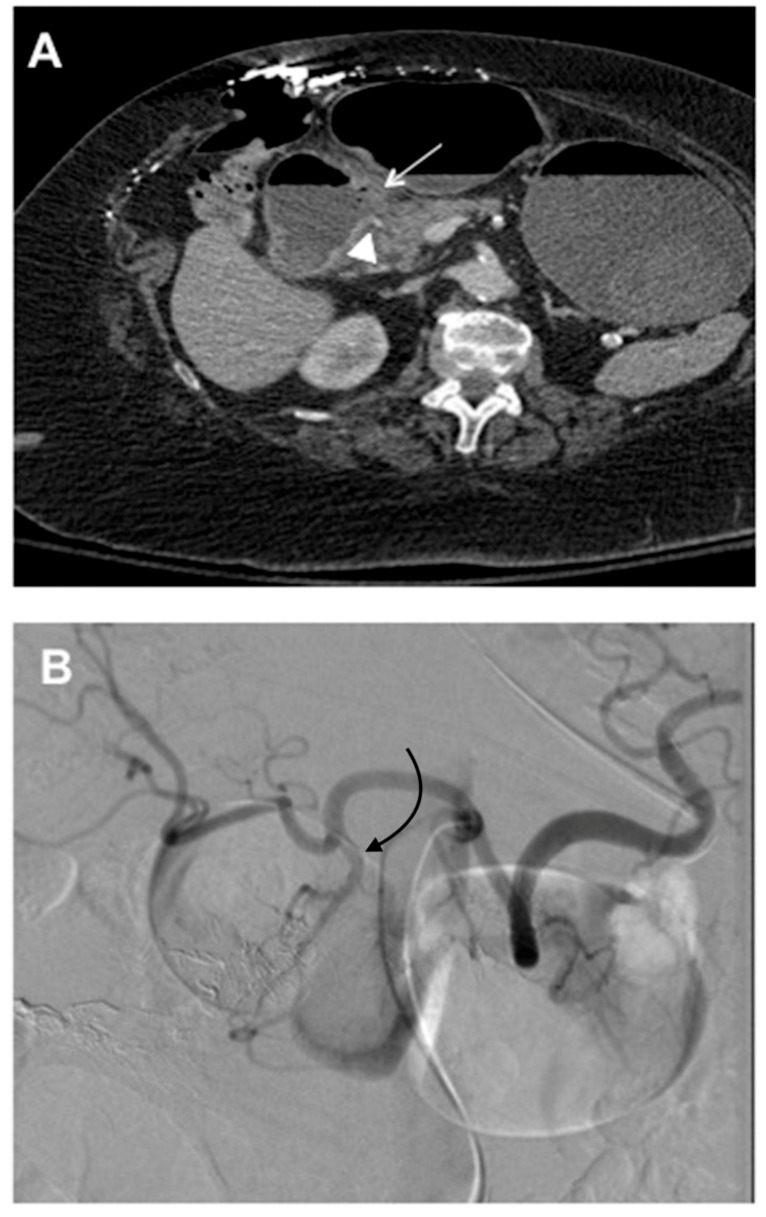

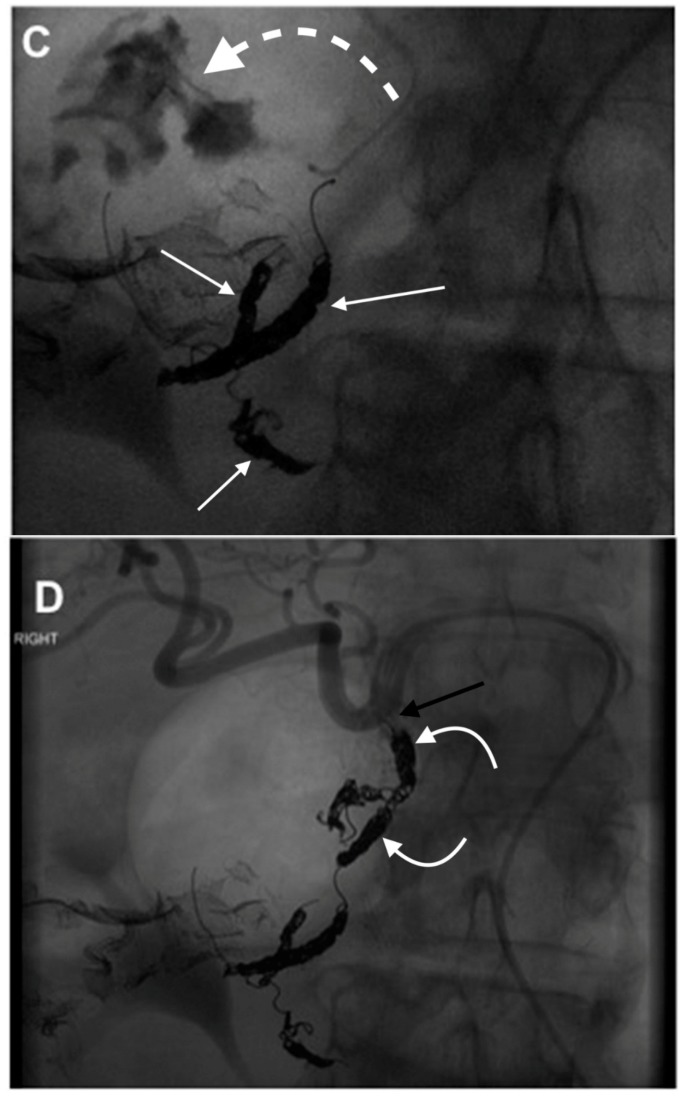
This 78 year old woman presented with melena, hematemesis, hypotension, and acute blood loss anemia. An endoscopy was not performed, given the high volume of blood which was felt to likely obscure the (**A**) Computerized Tomography (CT) images, demonstrating the duodenal perforation (white arrow) in the posterior wall with abnormal appearing GDA coursing directly adjacent to the perforation (white arrowhead). The (**B**) celiac angiogram shows the origin of the GDA (curved black arrow). The (**C**) branches of the GDA that were distal to the duodenal ulcer were coil emobolized (white straight arrows) and the repeat angiogram from the proximal to mid GDA, showed a contrast extravasation into the duodenum (curved dashed arrow). (**D**) Coil embolization (curved white arrows) was done across and proximal to the site of the extravasation up to the origin of the GDA from the common hepatic artery (black arrow).

**Figure 3:**
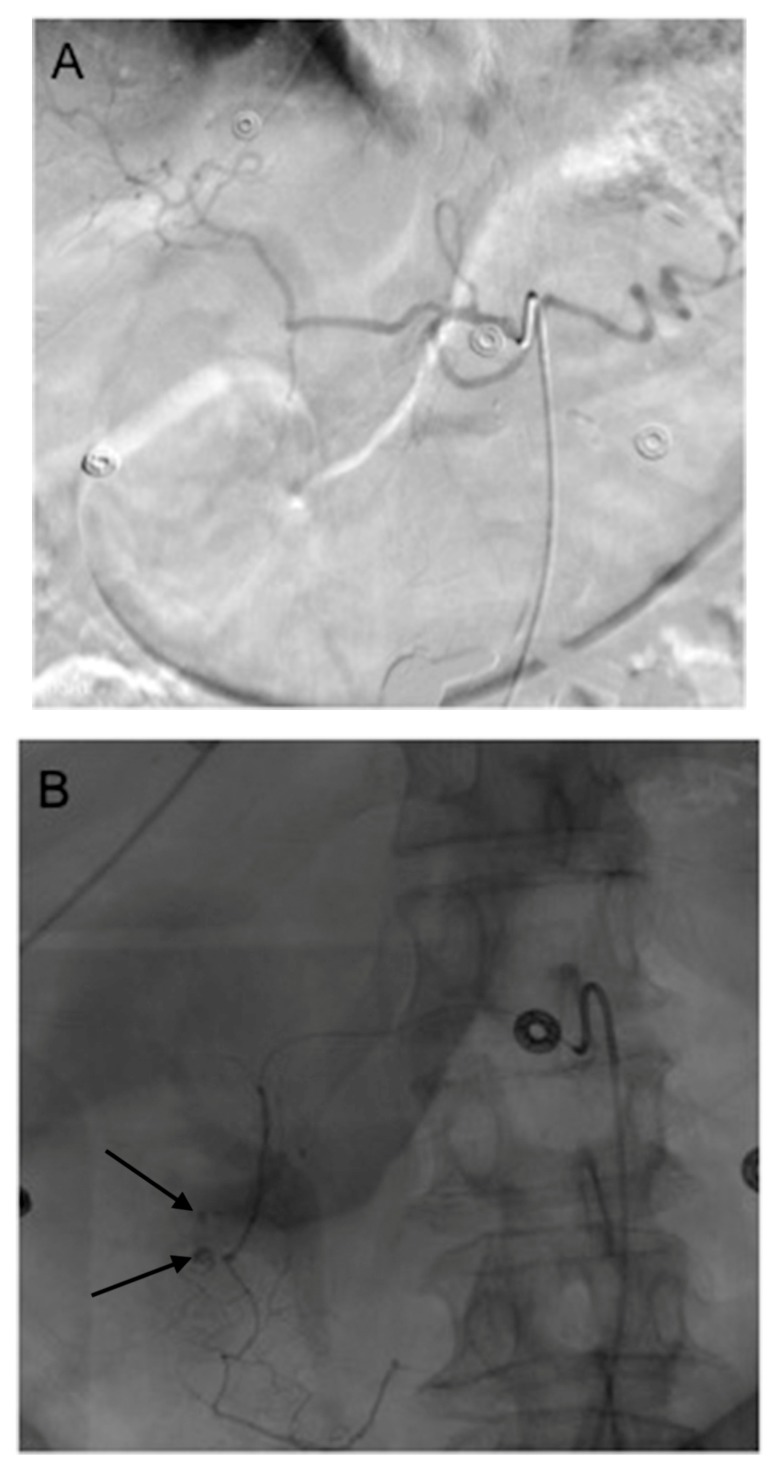

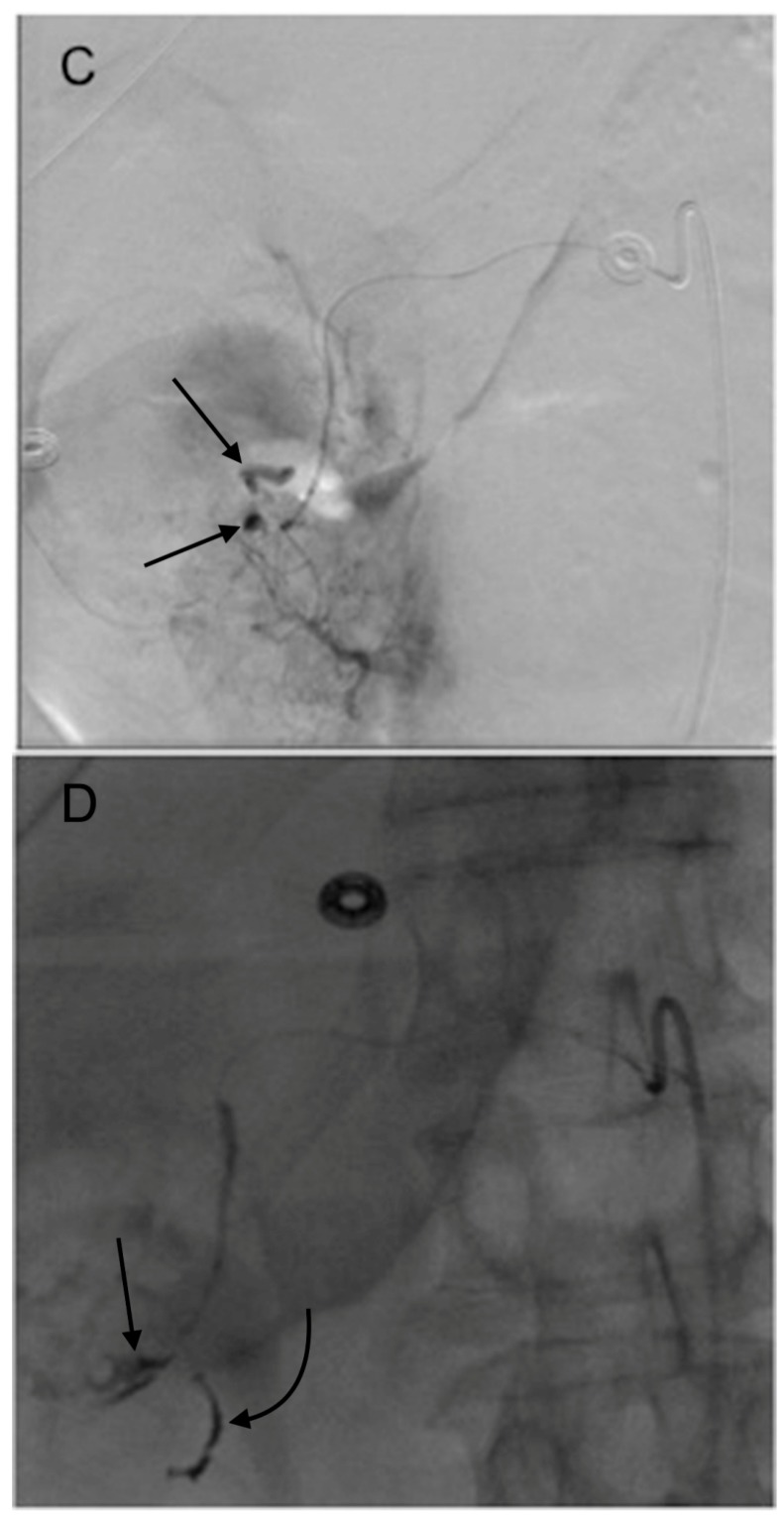

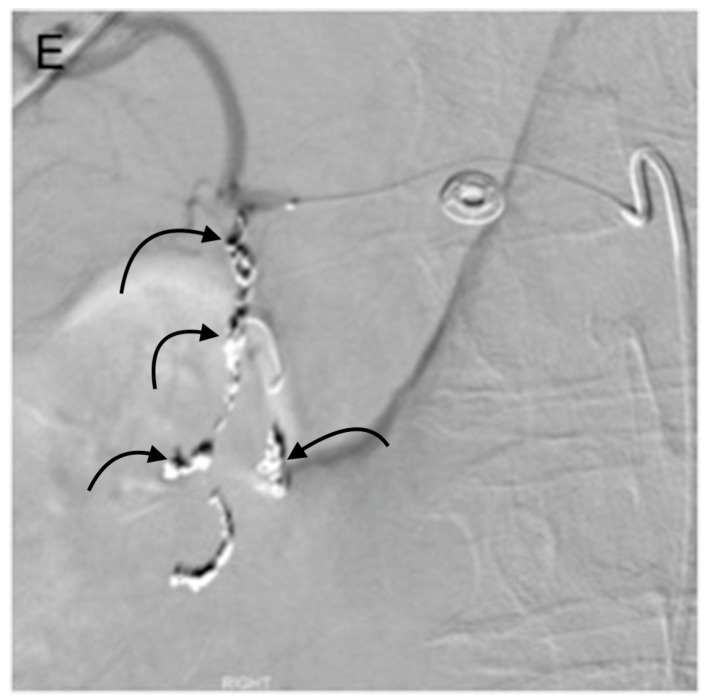
This 77 year old woman had a known history of multiple gastric and duodenal ulcers, and presented with massive upper gastrointestinal (GI) bleeding. A (**A**) celiac angiogram showed severely clamped down vessels that were consistent with hypotensive state and vasopressor use. The (**B**) native and (**C**) subtracted images show multiple sites of extravasation (black arrows). The coils embolization (black curved arrow) was done distal to the extravasation (black straight arrow) (**D**) and back to the origin of the GDA as it arose from the common hepatic artery (**E**). The bleeding was successful stopped.

The first transcatheter embolization for upper GI tract bleeding was performed by Josef Rösch in 1972 [12]. Since then, significant technological advances have been made, making endovascular management an integral part of the management of upper GI bleeding [21]. Given the dual supply to the duodenum from the celiac trunk (GDA), as well as the superior mesenteric artery (through the inferior pancreaticodoudenal arcades), embolization that is distal to the site, and proximal to the bleeding is needed for effective embolization (Figure 2 and Figure 3) [21].

The GDA embolization first starts with gaining arterial access, typically via the femoral or left radial artery. The celiac trunk, which arises from the anterior surface of the aorta at approximately the T12 level, is catheterized and a celiac angiogram is performed [21].

Depending on the angiographic findings, and whether the history and the site of bleeding are seen endoscopically, the selective catheterization of the GDA or left gastric artery is performed with a microcatheter and microwire. A superselective angiogram is performed and, depending on results, a targeted or empiric embolization is performed. Given the rich arterial collateral supply in the upper GI tract, embolization starts distal to most distal extravasation site and continues to the origin of GDA. This is done to prevent ‘backdoor’ and collateral supply to the site of bleeding.

The choice of embolics are variable and include gelatine sponge, particles (Embopheres^®^ Merritt Medical, Salt Lake City, UT, USA, or PVA particles), liquid agents e.g., *N*-butyl 2-cyanoacrylate (NBCA) or ethylene-vinyl alcohol copolymer (Onyx^®^, Micro Therapeutics, Inc., Irvine, CA, USA), AMPLATZER™ Vascular plugs (St. Jude Medical, Saint Paul, MN, USA) and coils [20,22].

The technical success of angiographic embolization for upper GI bleed is between 95–100%. Durable clinical success (no re-bleeding) is lower, ranging between 50% and 80% [19,23,24]. At the conclusion of a GDA embolization celiac trunk and superior mesenteric (SMA) angiograms are done. The latter is necessary to make sure no “back-door” filing of the site of bleed by collaterals such as the IPDA.

Rebleeding after endovascular embolization is explained by several factors. Rich collateral blood supply of the duodenum, including not only the GDA but also the pancreaticoduodenal, superior mesenteric artery branches, and gastroepiploic vessels which means that coiling of the gastroduodenal artery from the celiac axis alone may be inadequate to control duodenal blood supply and a duodenal ulcer might bleed through collateral supply from superior mesenteric artery [21].

Occasionally vasoconstriction critically ill and volume-depleted patients (see Figure 3), which in turn resolves after volume restoration and cessation of pressors expands the lumen of the vessel [20,25]. Coils sized during time of vasoconstriction may no longer able to occlude the vessel after expansion.

## 4. GDA Pseudoaneurysm Embolization

**Figure 4:**
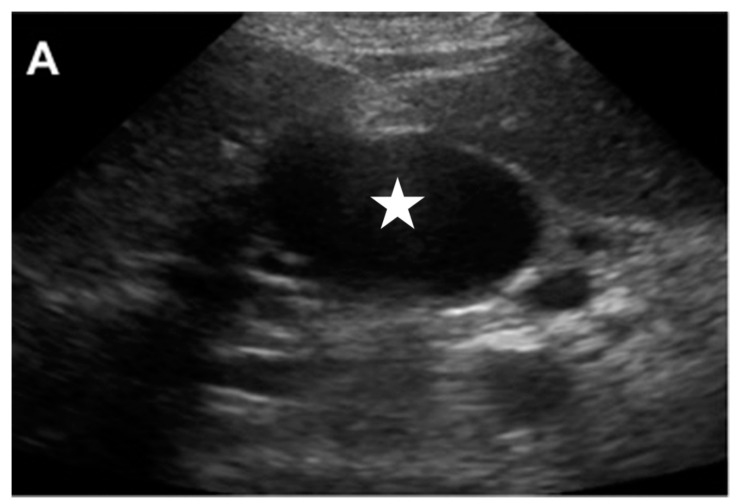

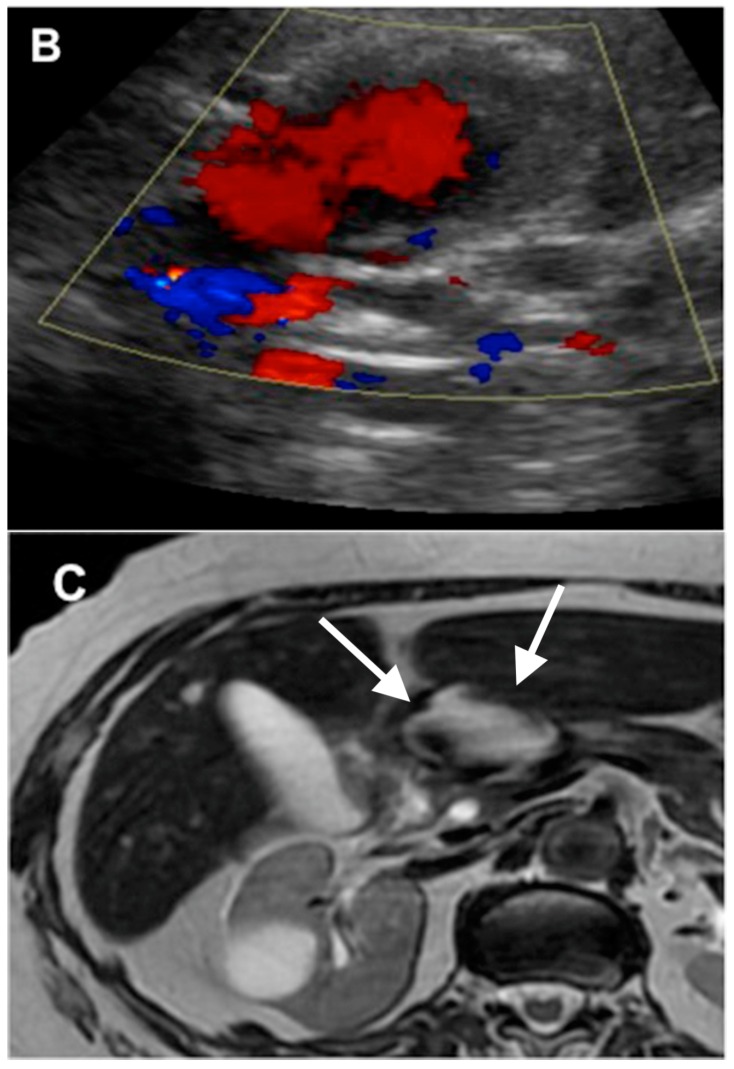

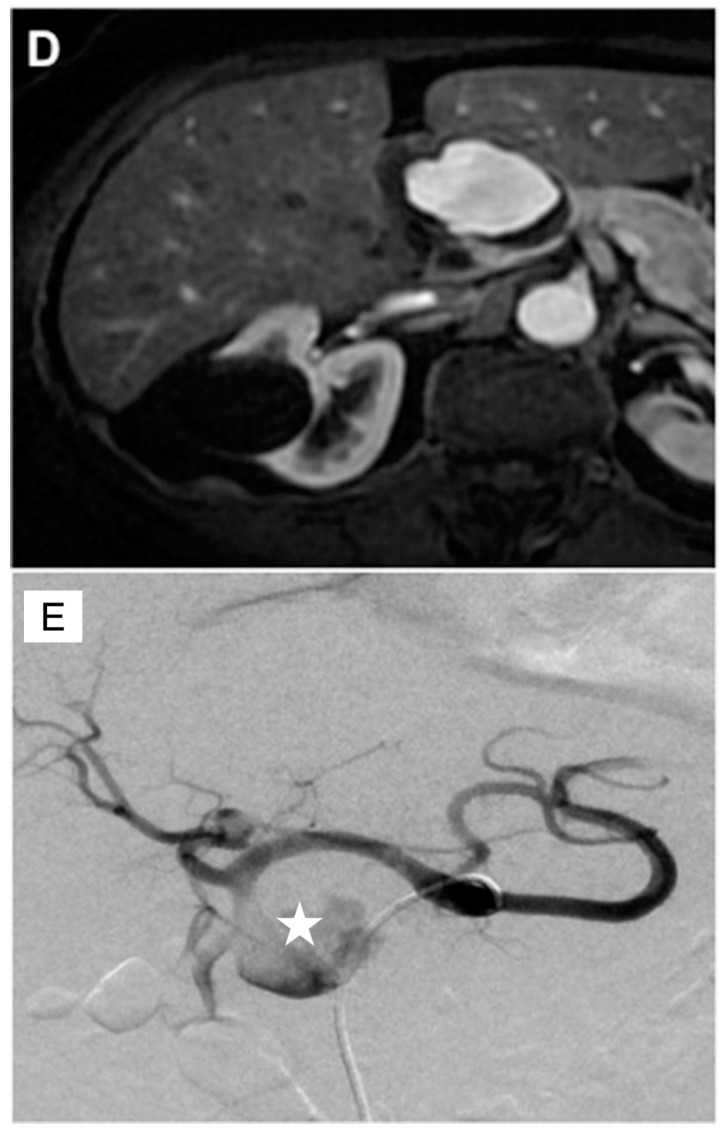

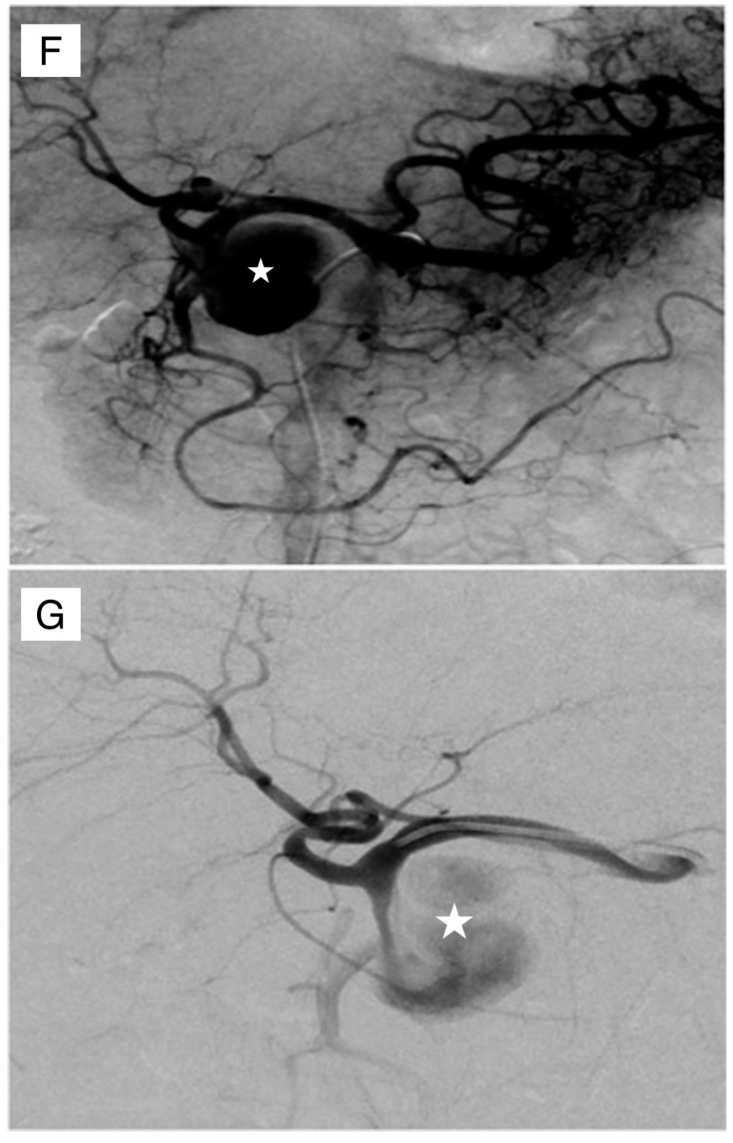

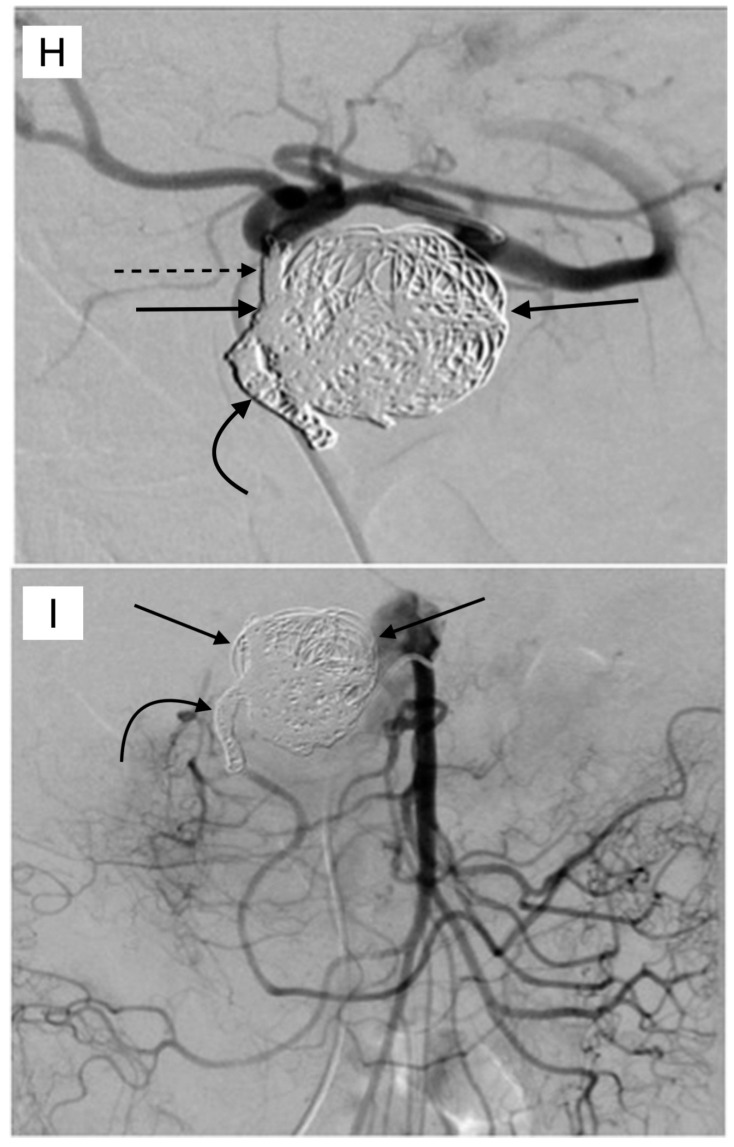
This 75 year old female with chronic pancreatitis secondary to pancreatic divisum who presented with worsening abdominal pain and early satiety. The (**A**) transverse abdominal ultrasound showing hypoechoic cystic structure (white star), separate from the gallbladder. The (**B**) color Doppler showed this to be highly vascular (aneurysm or pseudoaneurysm). The (**C**) T2 weighted abdominal magnetic resonance imaging (MRI) showed the mixed signal intensity within this aneurysm (white arrows). The (**D**) contrast enhanced imaging in the arterial phase showed the filling of this pseudoaneurysm with some peripheral thrombus. The patient was brought to angiography for an urgent angiogram and embolization. The celiac angiogram in the (**E**) early arterial and (**F**) parenchymal phase showed the large pseudoaneurysm (white star) arising from the GDA. The (**G**) common hepatic artery angiogram showed similar findings. The (**H**) embolization was then done. The coils were first placed distal to the pseudoaneurysm (curved black arrow), then within the pseudoaneurysm (straight black arrows), and then within the GDA that was proximal to the pseudoaneurysm (dashed black arrow). The (**I**) superior mesenteric (SMA) injection showed no backdoor filling of the pseudoaneursym. Again, the coils were seen distal to the sac (curved black arrow), as well as within the sac (straight black arrows).

Pseudoaneurysm of GDA is most commonly result from chronic pancreatitis and are seen in up to 20% of cases of chronic pancreatitis (Figure 4) [26]. Other common causes include postsurgical [27] or peptic ulcer disease [28]. They may be asymptomatic, present with intermittent bleed, present secondary to compression of the the common bile duct and/or duodenum or present with life threatening rupture [26]. When, GDA pseudoaneurysms rupture, the mortality is approximately 75% [29,30]. Rupture presents with hypotension, massive upper GI bleed, or retroperitoneal hemorrhage [31]. Given the high mortality with rupture, GDA pseduoaneurysms of any size should be treated. “Front door” and “back door” +/− sac embolization is required for effective embolization of pseudoaneurysms [21].

Historically, psuedoaneursyms were treated surgically, through ligation and/or resection. Transarterial embolization has now become the preferred treatment method in GDA pseudoaneurysms, given superior success rate and improved mortality, when compared to surgery [30]. The technical and clinical success is between 70 to 100% [32]. The potential complications are hemorrhage, reperfusion of the aneurysm sac via retrograde filling or through collaterals, or the migration of the coil [29,32,33].

Endovascular embolization can be utilized in the cases of hemobilia. The causes of hemobilia could include trauma (often iatrogenic), vasculitis, hepatobiliary tumors, and vascular malformations. Hemobilia presents with melena in 90% of cases, hematemesis in 60% of cases, biliary colic in 70% of cases, and obstructive jaundice in 60% of cases [34]. Angiographic embolization is now considered the first line therapy for hemobilia [35]. An abnormal connection between the common bile duct and gastroduodenal artery may cause extrahepatic hemobilia, which is most commonly caused by gastrodeudenal artery pseudoaneurysms [36]. The embolization of the gastroduodenal artery has been described as a safe method for the treatment of the hemobilia that is caused by gastroduodenal artery pseudoaneurysms [37].

## 5. Pre-Y-90 GDA Embolization

**Figure 5:**
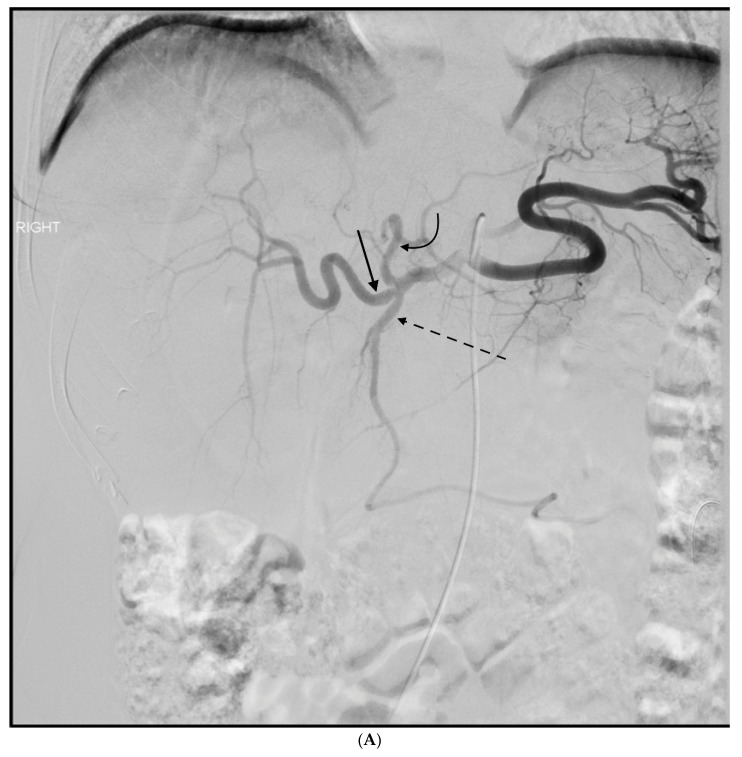

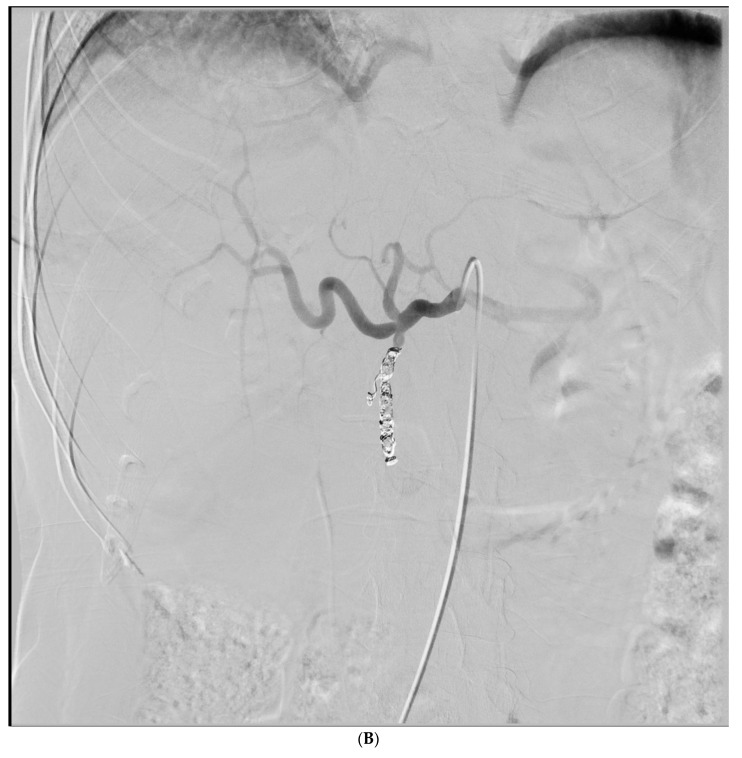
This 48 year old female with metastatic colo-rectal cancer was referred for selective internal radiation therapy, using Yittrium-90. The (**A**) mapping angiogram showed a trifurcation of the common hepatic artery into the GDA (dashed arrow), left hepatic (curved arrow), and right hepatic (straight arrow) arteries. GDA embolization with this anatomic configuration was felt to be necessary in order to reduce the chances of the non-target duodenal embolization. The (**B**) GDA was successfully cannulated with a microcatheter, and multiple coils were placed successfully, which embolized this vessel. The Yttrium-90 (Y-90) administration was subsequently performed without clinical or radiographic evidence of non-target embolization to the duodenum.

The coil embolization of GDA, prior to Y-90 treatment, is sometimes performed in order to prevent non-target embolization. Selective internal radiation therapy (SIRT) with Yttrium-90 (Y-90) is performed in order to provide oncologic control of liver tumors, including primary liver cancer, (hepatocellular carcinoma [HCC]) and for liver dominant metastases, such as in unresectable colorectal hepatic metastases. Commercially available resin or glass microspheres (SIRSpheres, Sirtex Medical Ltd., Lane Cove, Sydney, NSW, Australia, and TheraSphere, MDS Nordion, Ottawa, ON, Canada) contain yttrium, a β-emitting radioisotope, which leads to local irradiation [38]. Depending on the patient’s anatomy (Figure 5) and flow, the GDA may be coil embolized prior to Y-90 administration radiation therapy. Coil embolization of the GDA is done to prevent prevent the non-target embolization of the microspheres to the duodenum, which would lead to severe ulcerations [39]. Single photon-emission computed tomography (SPECT) with technetium macroaggregated (Tc-99 m) albumin (MAA) is performed after the coil embolization procedure, in order to evaluate for the presence of non-target embolization. The MAA particles are approximately the same size as microspheres therefore predicting non-target Y-90 microspheres [40].

Several studies showed that no difference was seen in the incidence of gastrointestinal complications in patients with prophylactic embolization of the gastroduodenal artery, when compared to the patients without embolization [41,42]. A few complications were reported when the microspheres were injected distally to the origin of these arteries, or even when the reversed flow of the GDA was present [41]. Consequentially, routine pre-Y-90 GDA embolization has fallen out of favor and is now only recommended if the pre-Y-90 MAA injection shows a significant duodenal uptake [39,41,42]. If the complete embolization of the GDA is undertaken, it should be embolized to the origin of the vessel, as the small, very proximal GDA branches may hypertrophy in response to the incomplete embolization [43]. The GDA embolization should be avoided, when there is retrograde flow secondary to celiac stenosis. One of the complications of GDA embolization is reopening of coiled or even newly developed hepatointestional collaterals. These collaterals are reported to have been found in up to 44% of patients, after the GDA occlusion [44]. In the majority of these cases, the SIRT could still be performed with the recoiling or modification of the microcatheter position. However, approximately 5% of the patients that were undergoing the GDA embolization of SIRT could not be performed upon because of the development of these collaterals [41]. Some of the studies support use of anti-reflux catheters, such as the Surefire ^®^ catheter (Westminster, CO, USA), as they may obviate the need for the coil embolization of the GDA all together [45].

## 6. Complications of GDA Embolization

The main complication after transarterial embolization is ischemia. Although the upper gastrointestinal tract has a rich collateral blood supply, ischemic complications can still occur in 7 to 16% of cases [46,47]. Previous foregut surgery and the use of either liquid (cyanoacrylate) or particulate embolics, are risk factors for ischemic complications [48]. An ischemic complication after embolization can present acutely, with mucosal ischemia and possible perforation or present in a delayed manner with ischemic duodenal stenosis [49]. Non-target embolization rarely results in ischemic pancreatitis [50].

## 7. Conclusions

Upper GI bleeding is a potentially life threatening condition with the overall mortality rates for non-variceal bleeding approaching 10% [1]. While endoscopic and medical therapies are largely effective, some patients are refractory to such management. In these cases, urgent endovascular embolization of the gastrodoudenal artery is potentially lifesaving. Ischemic complications can occur in up to 16% of embolizations [49]. Pseudoaneurysms of the GDA, which typically occur in the condition of pancreatitis, can be effectively treated with a transcatheter embolization. For both GI bleeding and pseudoaneurysm embolization, a distal and proximal embolization is necessary in order to prevent backdoor perfusion at the site of the bleeding or pseudoaneurysm. Prior to the Y-90 administration, the GDA embolization can be considered when the anatomy or flow patterns appear to be predisposed to a possible non-target GI embolization.
